# Isatin Schiff base is an effective corrosion inhibitor for mild steel in hydrochloric acid solution: gravimetrical, electrochemical, and computational investigation

**DOI:** 10.1038/s41598-022-22611-4

**Published:** 2022-10-22

**Authors:** Ahmed A. Al-Amiery, Waleed Khalid Al-Azzawi, Wan Nor Roslam Wan Isahak

**Affiliations:** 1 Department of Chemical and Process Engineering, Faculty of Engineering and Built Environment, Universiti Kebangsaan Malaysia (UKM), UKM, P.O. Box: 43000, Bangi, Selangor Malaysia; 2 Energy and Renewable Energies Technology Center, University of Technology-Iraq, Baghdad, 10001 Iraq; 3 Al-Farahidi University, Baghdad, 10001 Iraq

**Keywords:** Chemistry, Materials science

## Abstract

This paper describes the synthesis and characterisation of an isatin Schiff base, namely 2-(2-oxoindolin-3-ylidene) hydrazinecarbothioamide (OHB). The chemical structure of OHB was elucidated through proton-nuclear magnetic resonance (^1^H-NMR), carbon-nuclear magnetic resonance (^13^C NMR), and Fourier-transform infrared (FT-IR) spectroscopic techniques. OHB was evaluated for its corrosion inhibition ability on mild steel specimens in 1 M HCl using gravimetrical methods and electrochemical measurements such as electrochemical impedance spectroscopy (EIS) and potentiodynamic techniques complemented with microscopic analysis. The results indicated that OHB is a mixed-type inhibitor and showed good corrosion inhibition, with a maximum corrosion inhibition efficiency of 96.7% at a concentration of 0.5 mM and 303 K. The inhibition performance increased with an increasing OHB concentration and decreased with increasing temperature. The inhibition efficiency was attributed to the formation of a protective film on the surface of the tested mild steel coupon. The electrochemical impedance studies also indicated that the charge transfer resistance increased with an increase in OHB concentration. The morphological analysis confirmed the inhibition performance of OHB and the protective barrier film conformed to Langmuir monolayer adsorption. The experimental and theoretical corrosion kinetics and thermodynamic parameters were in agreement and revealed that an adsorption film of Fe–N coordination bonds formed on the mild steel surface.

## Introduction

Mild steel is often used in the oil and gas industry due to its reasonable price and excellent mechanical strength. Techniques such as oil well acidification, acid pickling, and acid descaling are performed using strong acids such as hydrochloric acid to remove unwanted scales and salt deposits and to enhance oil recovery^1^. However, these acids may corrode the surface of the mild steel resulting in significant costs for renovation and system maintenance, as well as physical and environmental losses^2^.

Corrosion inhibitors are classified according to their chemical nature, mode of action, and other properties, with the most popular type being organic corrosion inhibitors. Their popularity has grown due to their low cost, simplicity of installation, and high level of protection. They prevent corrosion by adsorption to the steel surface, thereby protecting the metal surface from corrosive solutions^3,4^ and the presence of heteroatoms such as phosphorus, sulfur, oxygen, nitrogen, etc. makes them effective in a variety of acidic solutions^5,6^. Direct contact between the mild steel and the corrosive environment is avoided by adsorption of the organic molecules onto the metal surface^7,8^, however, most organic corrosion inhibitors are expensive and hazardous to the environment and public health. Organic compounds with heterocyclic and aromatic heterocyclic rings have stronger inhibition effects in acidic solutions^9–13^. The adsorption of organic chemicals is determined by chemical (the formation of coordination bonds between the atoms of the atoms on the surface of the mild steel and the electron pairs of the inhibitor molecule) and physical bonding (the Van der Waals bonds between the inhibitor molecules and the iron charges). The effectiveness of the inhibitors can be explained by their strong polarizability and low electronegativity, which enable them to cover large metal surfaces and rapidly transfer electrons to empty atomic orbitals^14^. Organosulfur and nitrogen-containing inhibitors prevent the corrosion of steel in HCl and H_2_SO_4_ acids. Schiff bases are utilized as complexing agents in metal complexes and they have an azomethine  group. These chemicals are used to clarify the process of racemization reaction transition because they share structural similarities with neutral biological systems due to the presence of nitrogen and oxygen donor atoms. However, most current thiosemicarbazide derivatives only have one aromatic ring. Several factors, including the electronic structure and the effects of static hindrance and aromaticity, influence the inhibition potential, thus it is difficult to predict the ability of a new organic compound with a Schiff base group, aromatic, heterocyclic, and six heterocyclic atoms to inhibit corrosion. A novel aspect of this study was the facile and affordable synthesis of a new corrosion inhibitor for preserving mild steel in 1 M hydrochloric acid. This Schiff base derivative features high inhibition efficiency (96.7%), low inhibitor concentration, extended working time, and strong interactions between mild steel surface and inhibitor molecules. The novel isatin Schiff base 2-(2-oxoindolin-3-ylidene) hydrazinecarbothioamide (OHB) containing a Schiff base moiety, thiosemicarbazide, and another isatin was synthesised and characterised, then its corrosion protection of mild steel in a 1 M HCl solution was evaluated in this study.

## Experimental

### Materials

The materials were analytical reagents purchased from Sigma-Aldrich (Selangor, Malaysia) and they were used without additional processing. The purity of the chemicals was determined by TLC (thin-layer chromatography) on silica gel G plates with benzene, ethyl acetate, methanol 4 to 3 to 3 (volume to volume) or toluene: acetone 7.5 to 2.5 (volume to volume) as the mobile phase and locating the spots under UV illumination at 254.0 nm and 365.0 nm. The following equipment was used in this study, a Thermo Scientific Nicolate 6700 Fourier transform infrared (FT-IR) spectrometer (Thermo Fisher Scientific, Waltham, MA, USA) and an AVANCE III 600 MHz spectrometer (Bruker, Billerica, MA, USA).

### Inhibitor synthesis

**Figure 1:**
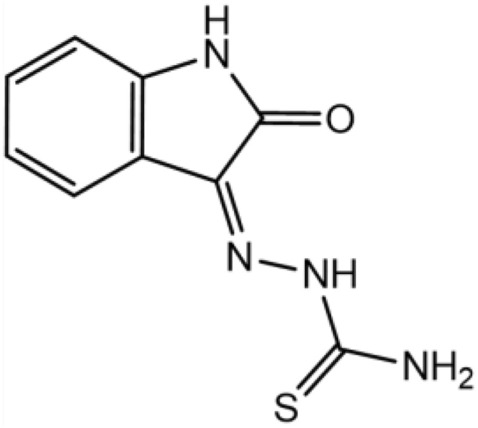
The chemical structure of OHB.

Equimolar quantities (0.03 mol) of isatin and thiosemicarbazide were dissolved in warm ethanol containing 1 ml of glacial acetic acid. The reaction mixture was refluxed for 8 h, concentrated and recrystallized using ethanol, yielding 88% OHB (Fig. 1), the purity of which was determined by TLC. The aluminium 60F-254 base of the thin layer chromatography plates (10.0 cm × 20.0 cm) was coated with silica to create a stationary phase that was roughly 0.50 mm thick and placed in a saturated chromatographic chamber with a solvent of methanol, ethyl acetate, and acetone (2.50 to 2.50 to 5.0). FT-IR: 3517.99, 3419.45, 3335.82, 3265.97 and 3164.04 cm^−1^ (NH–, NH, and NH_2_), 3063.49 cm^−1^ (C–H aromatic), 1701.60 cm^−1^ (carbonyl), 1672.21 cm^−1^ (CH=N–), 1063.78 and 1130.56 cm^−1^ (C–O–C sy and asy). 1H-NMR (DMSO) (δ, ppm): 9.002 (1H, s, N–H); 8.653 (1H, s, N–H); 6.915–6.935 (1H, dd, J = 7.9, 7.5, 1.3 Hz); 7.060–7.100 (1H, tt, J = 7.9, 1.3, 0.5 Hz); 7.328–7.370 (1H, tt, J = 8.6, 1.3, 0.5 Hz); 7.641–7.660 (1H, dd, J = 8.6, 7.5, 1.3 Hz). ^13^C-NMR (DMSO) (δ, ppm): 111.51 (1C, s), 120.43 (1C, s), 121.44 (1C, s), 122.86 (1C, s), 131.75 (1C, s), 132.53 (1C, s), 142.81 (1C, s), 163.10 (1C, s), 179.19 (1C, s).

### Mild steel and chemicals

The mild steel was supplied by the Company of Specimens and its elements are listed in Table 1. The mild steel samples were prepared per ASTM G1-03^15^, and a silicon carbide sheet was used to abrade the surface. Concentrated HCl (37%) was diluted with distilled water to prepare the 1 M hydrochloric acid solution.

**Table 1:** The chemical composition (wt%) of mild steel.

Fe	P	S	Al	Si	Mn	C
Balance	0.09%	0.05%	0.01%	0.38%	0.05%	0.21%

### Weight loss measurements

According to NACE TM0169/G31^16^, the mild steel specimens were exposed to 1 M HCl in the absence and presence of the inhibitor (0.1, 0.2, 0.3, 0.4, 0.5, and 1.0 mM) at 303 K, for 1, 5, 10, 24, and 48 h, then heated to 313, 323, or 333 K for 5 h. The samples were then handled according to ASTM standard G1-03. Continuing the computation, the corrosion rate was measured using the mean weight loss^15^. Equations (1) to (3) were used to calculate the corrosion rate (C_R_), the inhibition efficiency (IE), and the surface coverage ($\theta$):

1 $${C}_{R }(mg\cdot {cm}^{-2}\cdot {h}^{-1})=\frac{W}{at}$$

2 $$IE\%=[1 - \frac{{C}_{R(i)}}{{{C}_{R}}_{o}}]\times 100$$

3 $$\theta =1 - \frac{{C}_{R(i)}}{{{C}_{R}}_{o}}$$

where w is the weight loss of mild steel sample (mg), $a$ is the tested coupon surface area (cm^2^), $t$ is the immersion time (h).

### Electrochemical analysis

For the duration of the test, the coupons were used as working electrodes and were cleaned as per ASTM G1-03^15^. The experiments were conducted at inhibition concentrations of 0.1 to 0.5 M in 1.0 M HCl that was vented but not agitated at 303 K, and the effective test area was 4.5 cm^2^. All experiments were performed in triplicate on a Gamry Instrument Potentiostat/Galvanostat/ZRA type REF 600, and the mean was computed using DC105 and EIS300 software. The dynamic current potential was altered from 0.25 to + 0.25 V SCE at a scan rate of 0.5 mVs1. All impedance values were fitted to the proper equivalent circuits (ECs) for the Gamry Echem Analyst program. An electrode of saturated calomel was used as the reference electrode in a Gamry water-jacketed glass cell with three electrodes: the working electrode, the counter electrode, and the corrosion inhibitor (SCE). To maintain the steady-state potential, electrochemical tests were started 30 min after the working electrode was immersed in the HCl solution^17–19^.

### Surface scanning electron microscopy

The corrosion action of the corrosive medium on the coupons after 5 h of treatment in the absence and with the presence of 0.5 mM OHB was evaluated by scanning electron microscopy on a Zeiss MERLIN Compact FESEM at the UKM Electron Microscopy Unit.

### Computations

Gaussian 09 was used to run the quantum chemistry calculations^20^. The inhibitor structure in the gas phase was optimized (d,p) utilizing the B3LYP technique and the basis set of “6-31G^++^”. The ionization potential ($I$) and electron affinity ($A$) correspond to ${E}_{HOMO}$ and ${E}_{LOMO}$, respectively, according to Koopman's theorem^21^. Equations (4) and (5) were used to compute each ionization potential and electron affinity:

4 $$I=-{E}_{HOMO}$$

5 $$A=-{E}_{LOMO}$$

Equations (6) to (8) were used to derive the electronegativity ($\chi$), softness ($\sigma$) and hardness ($\eta$):

6 $$\chi =\frac{I+A}{2}$$

7 $$\eta =\frac{I - A}{2}$$

8 $$\sigma ={\eta }^{-1}$$

Equation (9) was used to compute the fractional number of transferred electrons ($\Delta N)$,^21^:

9 $$\Delta N=\frac{{\chi }_{Fe} - {\chi }_{inh}}{2\left({\eta }_{Fe}+{\eta }_{inh}\right)}$$

Thus, ${\chi }_{Fe}$ and ${\chi }_{inh}$ indicate the electronegativities of iron and the studied inhibitor, correspondingly, whereas ${\eta }_{Fe}$ and ${\eta }_{inh}$ represent the hardness of iron and the investigated inhibitor. The ΔN value for the tested coupon (Fe) was calculated using Eq. (10), where ${\chi }_{Fe}$ = 7 eV, ${\eta }_{Fe}$ = 0 eV:

10 $$\Delta N=\frac{7 - {\chi }_{inh}}{2\left({\eta }_{inh}\right)}$$

A reactivity description called electrophilicity (Eq. 11) enables a quantitative assessment of a compound's overall electrophilic character on a universal scale, which is described as a measurement of energy reduction caused by the maximum electron flow between the donor and acceptor:

11 $$\upomega ={\chi }^{2}\times \left(0.5{\eta }^{-1}\right)=0.5\sigma {\chi }^{2}$$

## Results and discussion

### Synthesis

**Figure 2:**
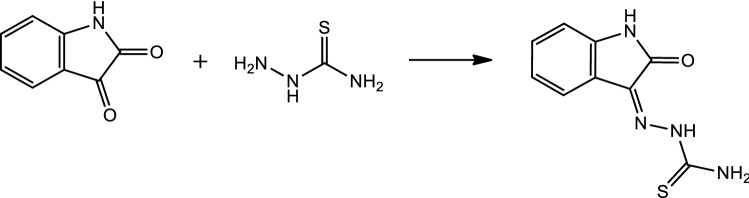
Isatin Schiff (OHB), chemical synthesis.

The chemical process shown in Fig. 2 was used to synthesize isatin Schiff, a corrosion inhibitor, using isatin and thiosemicarbazide.

**Figure 3:**
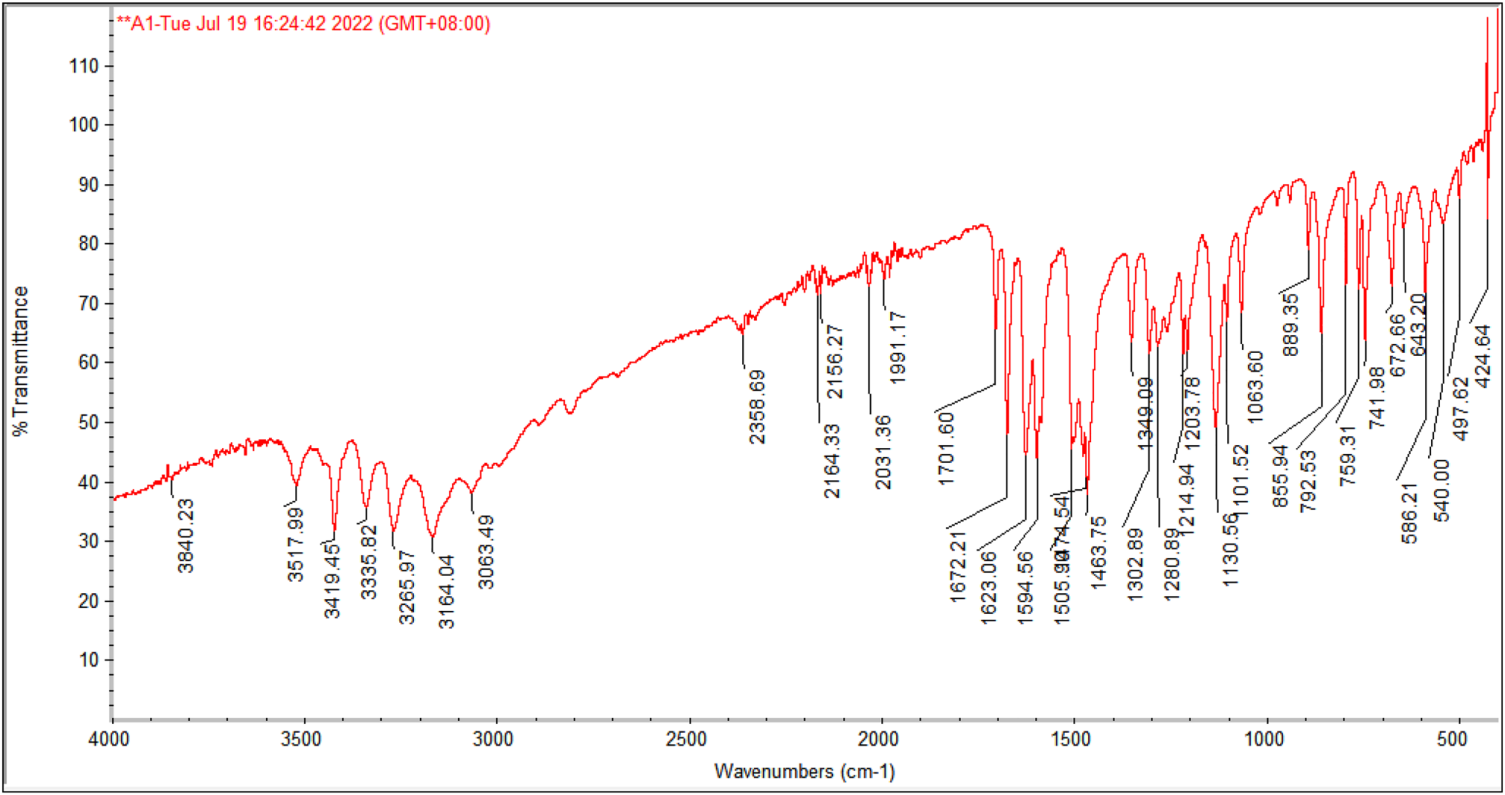
Fourier transform infrared spectrum of isatin Schiff (OHB).

Reflux of isatin with thiosemicarbazide in ethanol with the addition of a few drops of acetic acid was conducted in order to synthesize the target corrosion inhibitor. The corrosion inhibitor has a molecular weight of 188, which was determined using the chemical formula (C9H8N4O) and confirmed by CHN analysis. The Fourier transform infrared spectrum of this compound contains absorption bands for the NH–, NH, and NH2 groups at 3517.99, 3419.45, 3335.82, 3265.97, and 3164.04 cm^−1^, as well as the stretching carbonyl group at (1701.60 cm^−1^). The band at 1672.21 cm^−1^ of CH=N–, while the band at 3063.49 cm^−1^ is C–H aromatic (Fig. 3).

**Figure 4:**
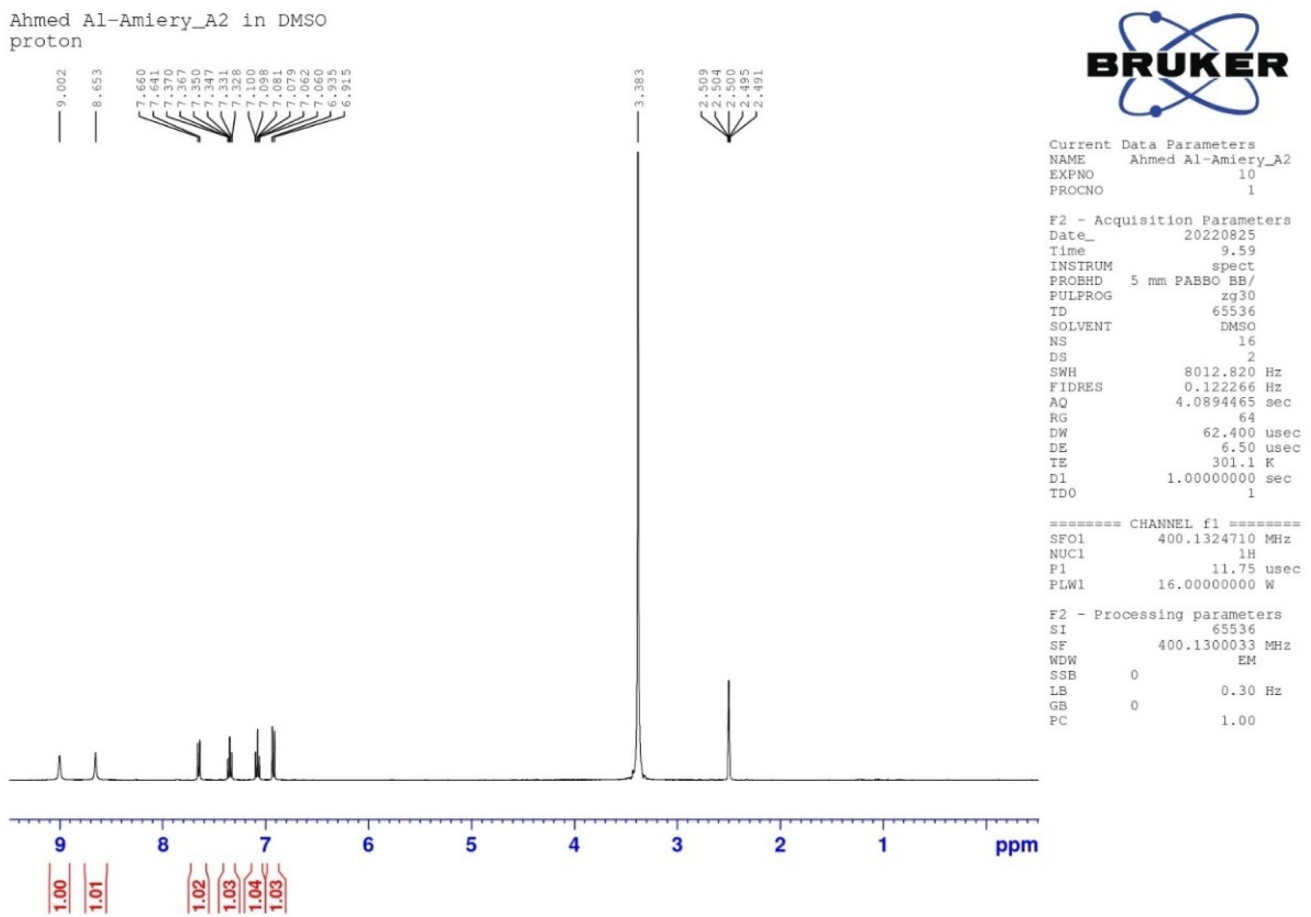
Proton nuclear magnetic resonance spectrum of isatin Schiff (OHB).

The ^1^HNMR spectrum exhibits a singlet at δ 9.002 ppm due to the NH as well as 8.653 for the second NH proton and a signal at δ 7.660 (dd, *J* = 8.7, 7.4 Hz, 1H) for aromatic proton. The signals at 7.370–328 (ddd, *J* = 8.7, 1.1, 0.5 Hz) and 7.100–7.060 (ddd, *J* = 8.8, 1.4, 0.5 Hz) are for aromatic protons. Finally, doublet-doublet signals at 6.935 and 6.915 (dd, 1H, *J* = 8.8, 7.4, 1.1 Hz) are for aromatic proton as shown in Fig. 4.

**Figure 5:**
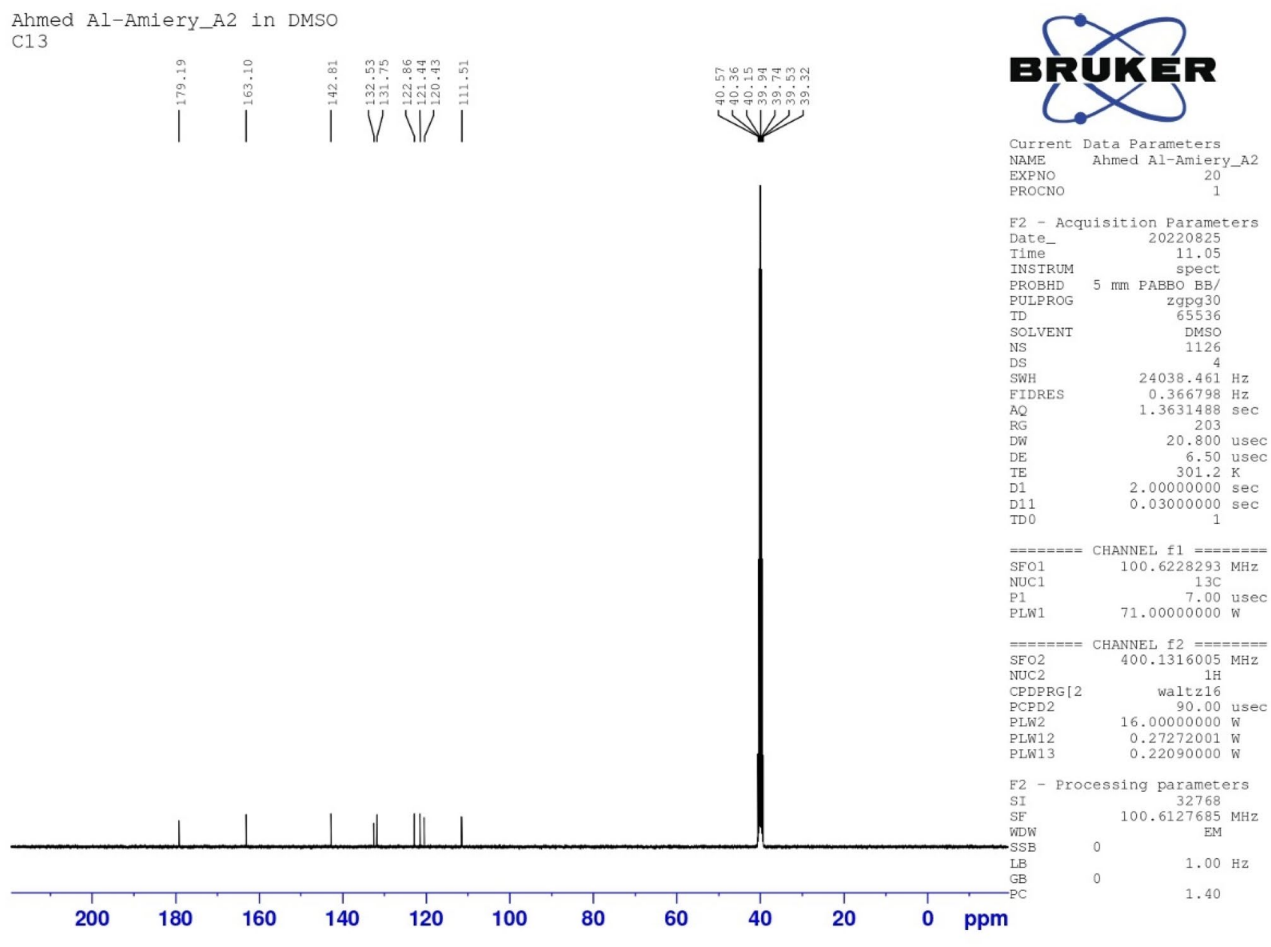
Carbon-*nuclear magnetic resonance* spectrum of isatin Schiff (OHB).

From 13C-NMR, a band appears at 179.19 ppm due to the thionyl group and a band at 163.10 ppm for carbonyl group whereas the signal at 142.83 for an imine (C=N) whereas the bands at 111.51, 120.43, 121.44, 122.86, 131.75, 132.53 ppm are from carbon atoms of the aromatic ring (Fig. 5).

### Weight loss investigations

**Figure 6:**
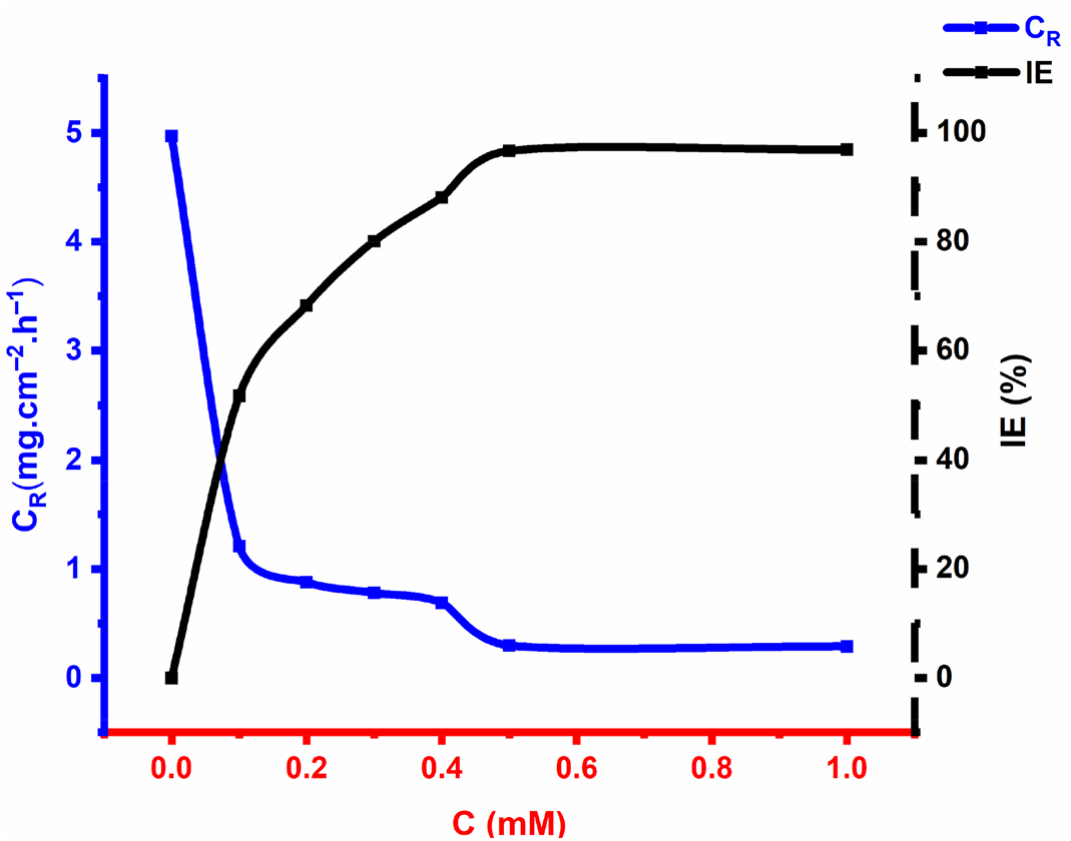
Different concentrations of OHB on the corrosion rate and anticorrosion efficacy of mild steel exposed to a 1 M corrosive environment for 5 h at 303 K.

A summary of the weight loss of steel samples in 1 M HCl solution in the absence and presence of OHB is shown in Fig. 6, demonstrating that the OHB shields the coupon surface from corrosion and that the corrosion protection ability increases with increasing OHB concentration. When the inhibitor concentration increased, the rate of corrosion reduced for a 5-h exposure, with the maximum inhibition (96.7%) at 0.5 mM OHB. The increase in the surface area caused by the adsorption of the inhibitor onto the surface of the mild steel may block the active sites and protect the surface of the mild steel from the corrosive medium. Since the OHB has a sizable molecular structure and contains many heterogeneous atoms (four nitrogen atoms in addition to one oxygen and one sulfur atom), it is believed that the binding of OHB molecules to the mild steel surface is responsible for the strong inhibition performance^15^.

As the studied inhibitor molecules were adsorbed onto the coupon surface to form a protective layer, the anti-corrosion efficacy of the inhibitor increased with increasing concentration up to 0.5 mM, after which, the inhibitory efficiency remained constant.

### The effect of exposure time

**Figure 7:**
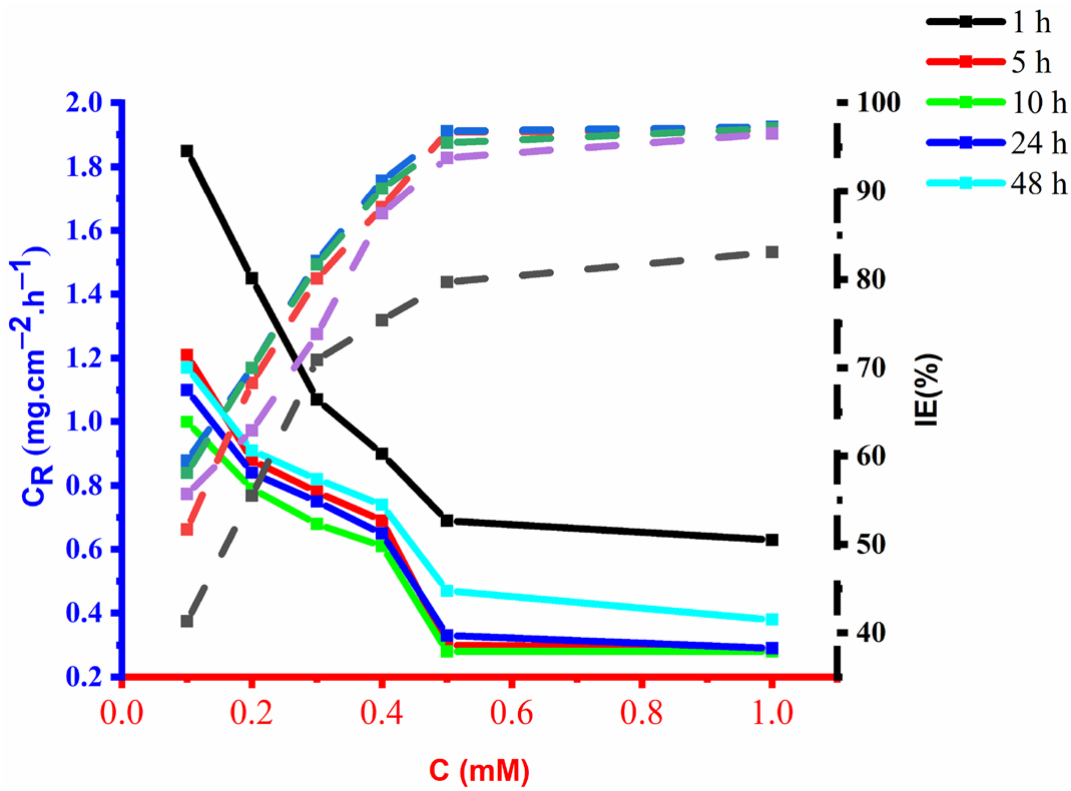
Different concentrations effect of OHB on the corrosion rate and anticorrosion efficacy of mild steel exposed to 1 M corrosive environment for 1–48 h at 303 K.

To determine the effect of immersion time on the efficacy of OHB corrosion inhibition, mild steel was immersed in the corrosive solution for 1 h to 48 h at 303 K (Fig. 7). The inhibitory efficacy improves rapidly with increasing immersion time up to 10 h, then gradually decreases from 10 to 24 h, and then more rapidly from 24 to 48 h. The increased exposure time increases the inhibitory efficiency by increasing the amount of OHB (due to the increased concentration) adsorbed onto the mild steel surface. Moreover, because many inhibitor molecules are adsorbed on the mild steel surface, the adsorption density of the inhibitor increases allowing physisorption (physical adsorption via Van Der Waals Forces) and chemical adsorption (chemosorption through the formation of coordination interactions between inhibitor molecules and iron atoms on the coupon surface). The effective area covered by the inhibitor may be reduced, thus the inhibitory activity may decrease if some inhibitor molecules escape the surface. The stability of the adsorbed inhibitor film in the presence of 1 M HCl solution is demonstrated by the relatively high inhibition efficiency that was observed during the prolonged exposure time.

The inhibitory efficiency of OHB is higher than that of certain chemical inhibitors documented in the literature^22–32^ for the concentration range under investigation (see Table 2). The target inhibitor OHB shows significantly better inhibitory efficiency at very low doses compared to the previously examined synthetic organic corrosion inhibitors.

**Table 2:** Compares reported corrosion inhibitors to the one under investigation.

Corrosion inhibitor	Metal	Acid	IE%	Refs.
OHB	Mild steel	HCl	96.7	–
3-(4-ethyl-5-mercapto-1, 2, 4-triazol-3-yl)-1-phenylpropanone (EMTP)	Mild steel	HCl	97	^22^
2-(4-phenyl-1H-1,2,3-triazol-1-yl) acetohydrazide	Mild steel	HCl	95.3	^23^
2-Amino-4-phenyl-N-benzylidene-5-(1,2,4-triazol-1-yl)thiazole	Mild steel	HCl	95	^23^
7-((1-benzyl-1H-1,2,3-triazol-4-yl)methyl)-1,3-dimethyl-3,7-dihydro-1H-purine-2,6–dione	Mild steel	HCl	91.7	^18,24^
7-((1-(4-fluorobenzyl)-1H-1,2,3-triazol-4-yl)methyl)-1,3-dimethyl-3,7-dihydro-1H-purine-2,6-dione	Mild steel	HCl	86.9	^24^
7-((1-(4-chlorobenzyl)-1H-1,2,3-triazol-4-yl)methyl)-1,3-dimethyl-3,7-dihydro-1H-purine-2,6-dione	Mild steel	HCl	94	^24^
7-((1-(4-bromobenzyl)-1H-1,2,3-triazol-4-yl)methyl)-1,3-dimethyl-3,7-dihydro-1H-purine-2,6-dione	Mild steel	HCl	91.8	^24^
7-((1-(4-iodobenzyl)-1H-1,2,3-triazol-4-yl)methyl)-1,3-dimethyl-3,7-dihydro-1H-purine-2,6-dione	Mild steel	HCl	90.9	^24^
5-methyl-4-((3-nitrobenzylidene) amino) -2,4-dihydro- 3H-1,2,4-triazole-3-thione	Mild steel	HCl	89.7	^25^
3-phenyl-4-amino-5-mercapto-1,2,4-triazole	Mild steel	HCl	97	^26^
2[5-(2-Pyridyl)-1,2,4-triazol-3-yl phenol	Mild steel	HCl	96.8	^27^
3,5-Bis(4-methyltiophenyl)-4H-1,2,4-triazole	Mild steel	HCl	93.5	^27^
3,5-Bis(4-pyridyl)-4H-1,2,4-triazole	Mild steel	HCl	89.1	^27^
3,5-Diphenyl-4H-1,2,4-triazole	Mild steel	HCl	82.8	^28^
3,5-Di(*m*-tolyl)-4-amino-1,2,4-triazole	Mild steel	HCl	95.8	^29^
5-Amino-1,2,4-triazole	Mild steel	HCl	24	^29^
5-Amino-3-mercapto-1,2,4-triazole	Mild steel	HCl	82	^29^
5-Amino-3-methyl thio-1,2,4-triazole	Mild steel	HCl	82	^29^
1-Amino-3-methyl thio-1,2,4-triazole	Mild steel	HCl	63	^30^
3-Benzylidene amino-1,2,4-triazole phosphonate	Mild steel	HCl	56.9	^30^
3-*p*-Nitro-benzylidene amino-1,2,4-triazole phosphonate	Mild steel	HCl	69.2	^30^
3-Salicylialidene amino-1,2,4-triazole phosphonate	Mild steel	HCl	43.2	^30^
3,5-Bis(methylene octadecyldimethylammonium chloride)-1,2,4-triazole	Mild steel	HCl	98.3	^31^
3-Amino-1,2,4-triazole-5-thiol	Mild steel	HCl	97.8	^32^

### The effect of temperature

**Figure 8:**
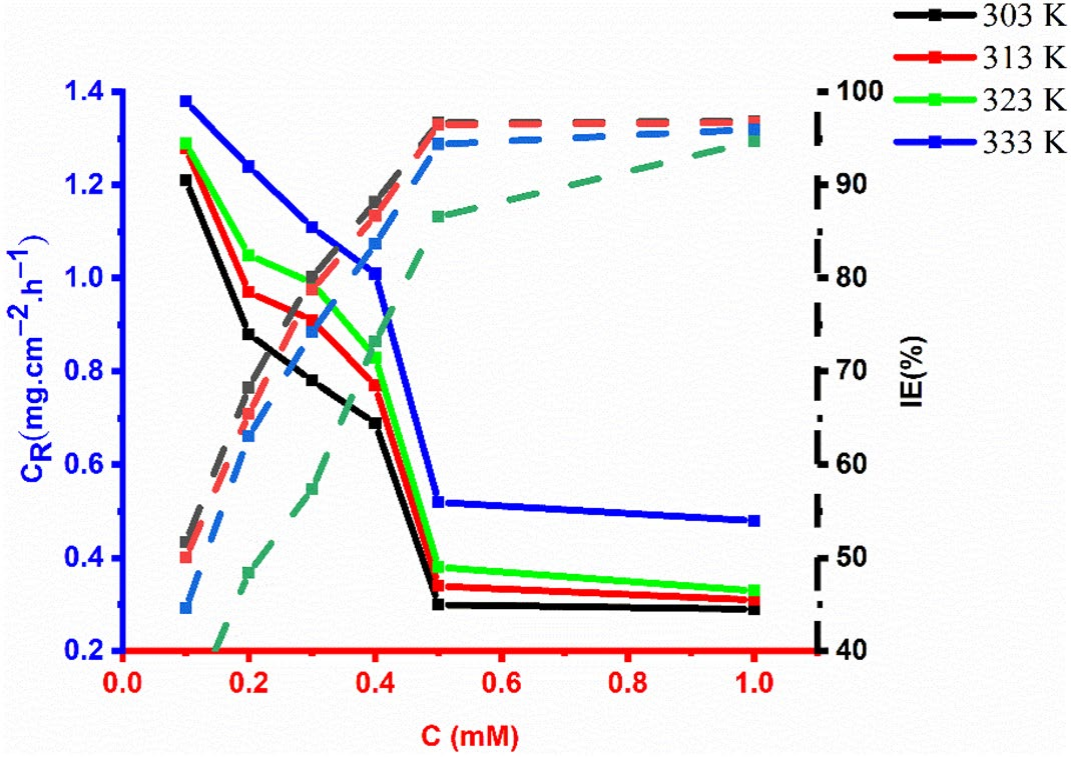
Different concentrations effect of OHB on the corrosion rate and anticorrosion efficacy of mild steel exposed to 1 M corrosive environment for 5 h at 303–333 K.

The anticorrosion inhibition efficiency of various OHB concentrations (0.1–1.0 mM) on mild steel immersed in corrosive solutions was investigated using the weight reduction technique after 5 h of immersion at various temperatures (303–333 K). The corrosion rate increased with temperature at the same inhibitor concentrations (Fig. 8), while anticorrosion efficiency decreased as temperature increased from 303 to 333 K. The OHB performed best at normal temperatures. The decrease in inhibitory potency with increasing temperature at all concentrations indicates physisorption. Furthermore, desorption occurs at high temperatures, which results in the loss of OHB molecules from the coupon surface.

The inhibition efficiency decreases due to an increased corrosion rate as the temperature rises because etching and inhibitor desorption occurs, increasing the amount of metal surface that comes into contact with the acidic solution and thus the rate of corrosion. The Arrhenius equation^33^, which is given by Eq. (12), is the best representation of the apparent activation energy of the corrosion process and corrosion rate:

12 $${C}_{R}=A\mathrm{exp}\frac{{-E}_{a}}{RT}$$

where, ${C}_{R}$ is the rate of corrosion, $R$ is the constant of gas, $T$ is the temperature and $A$ refers to the parameter of pre-exponential.

**Figure 9:**
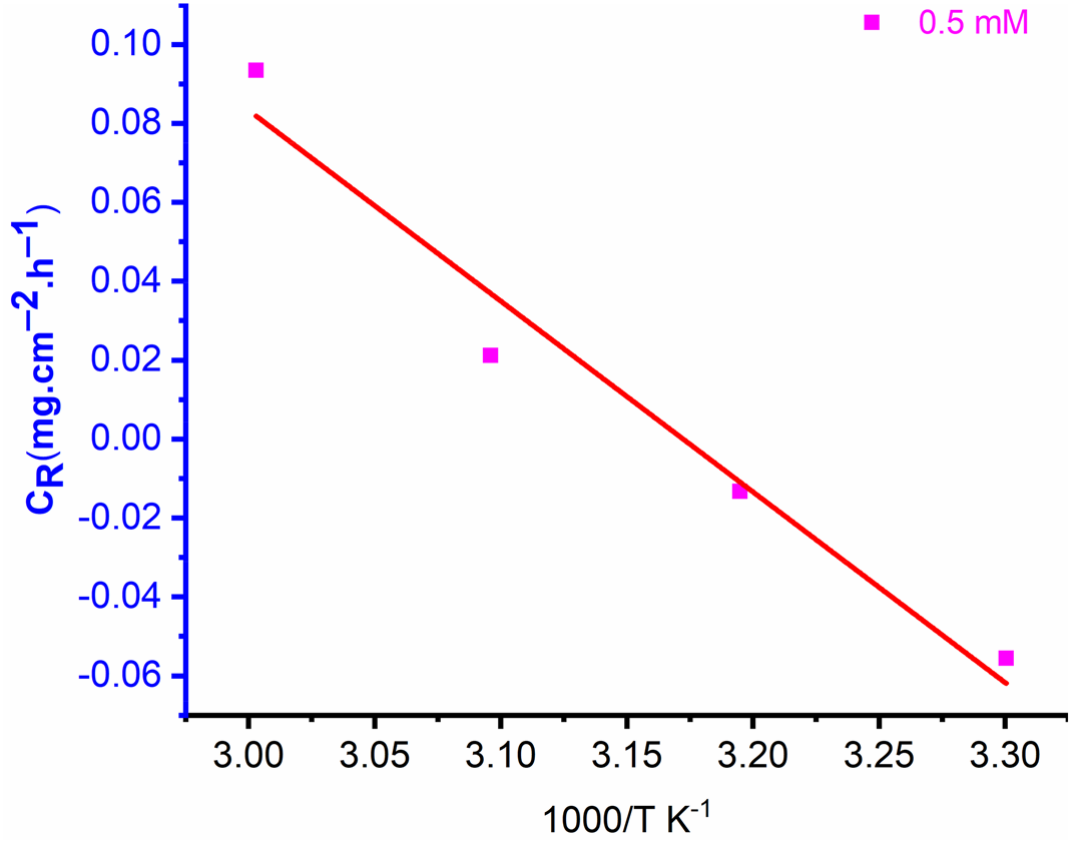
The Arrhenius plot of 〖log⁡C〗_R versus 1000/T for mild steel in 1 M HCl environment with the 0.5 mM of OHB.

The Arrhenius plot of $${\mathrm{log}C}_{R}$$ versus $$1000/T$$ for mild steel with the optimal concentration of OHB in a corrosive environment is demonstrated in Fig. 9. The activation energy ($${E}_{a})$$ value was estimated based on the slope $${(E}_{a}/2.303\mathrm{R})$$ and was equal to $${69.83 kJmol}^{-1}$$. According to the results, the activation energy for the uninhibited solution $${(29.11 kJmol}^{-1})$$ is less than that of the inhibited medium, indicating the formation of a protective film of inhibitor molecules on the surface of mild steel that increases the energy barrier for charge and mass transfer reaction^34^. The reduction in protection performance of OHB with the rising temperature indicates physisorption.

Using the modified Arrhenius relation (Eq. 13), The kinetic parameters are assessed in response to the variation in corrosion rate (C_R) with temperature.

13 $${C}_{R}=\frac{RT}{Nh}exp\left(\frac{{\Delta S}^{*}}{R}\right)exp\left(\frac{{-\Delta H}^{*}}{RT}\right)$$

where, h is the constant of Plank, N is the number of Avogadro, ∆S* is the activation entropy and ∆H* is the activation enthalpy.

**Figure 10:**
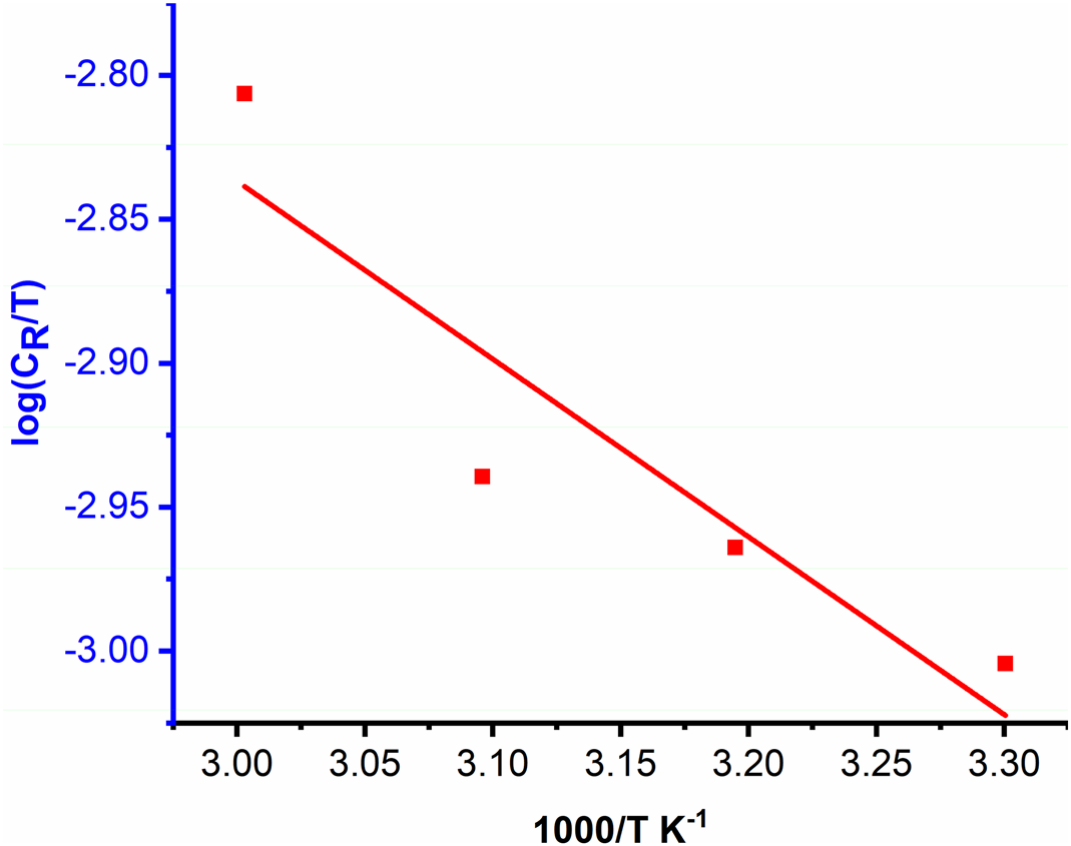
Arrhenius plot of log (C_R_/T) vs. 1000/T in the presence of 0.5 M OHB.

The entropy of activation and enthalpy of activation was determined from the intercept and slope, respectively, of the plot of the $\mathrm{log }({\mathrm{C}}_{R}/\mathrm{T})$ vs (1 $/\mathrm{T}$) in Fig. 10. Generally, chemisorption of the inhibitor molecules is predicted when the calculated $\Delta H$ for inhibited samples ($\Delta H=43.11 k.J.{mol}^{-1}$) are almost equal to that determined for the uninhibited $(\Delta H=40.36 k.J.{mol}^{-1}$) system^35^. As a result, observed activation parameters confirm the conclusion reached from adsorption parameters that, chemical adsorption dominates over physical adsorption at greater inhibitor concentrations. The observed slowing of the rate of corrosion at the optimal concentration can be explained by a significant reduction in the Arrhenius frequency factor ($A$).

At a low concentration of the inhibitor, the surface of mild steel is not entirely covered, so physical adsorption dominates over chemical adsorption providing a higher energy barrier for charge transfer. However, in the absence of the inhibitor, the ∆S* is negative (104.95 J K^−1^ mol^−1^) and in the presence of tested inhibitor at optimum concentration the ∆S* is also negative (123.82 J K^−1^ mol^−1^). This mechanism involves a delayed electrochemical discharge reaction, followed by an electrochemical desorption reaction (at a larger overpotential range, Eqs. (14–16))^36^:

14 $${H}_{3}{O}^{+}+M+{e}^{-}\to {MH}_{ads}+{H}_{2}O$$

15 $${2MH}_{ads}\leftrightarrow {H}_{2}+2M$$

16 $${MH}_{ads}{+H}_{3}{O}^{+}{e}^{-}\leftrightarrow {H}_{2}+M+{H}_{2}O$$

### Adsorption isotherm

**Figure 11:**
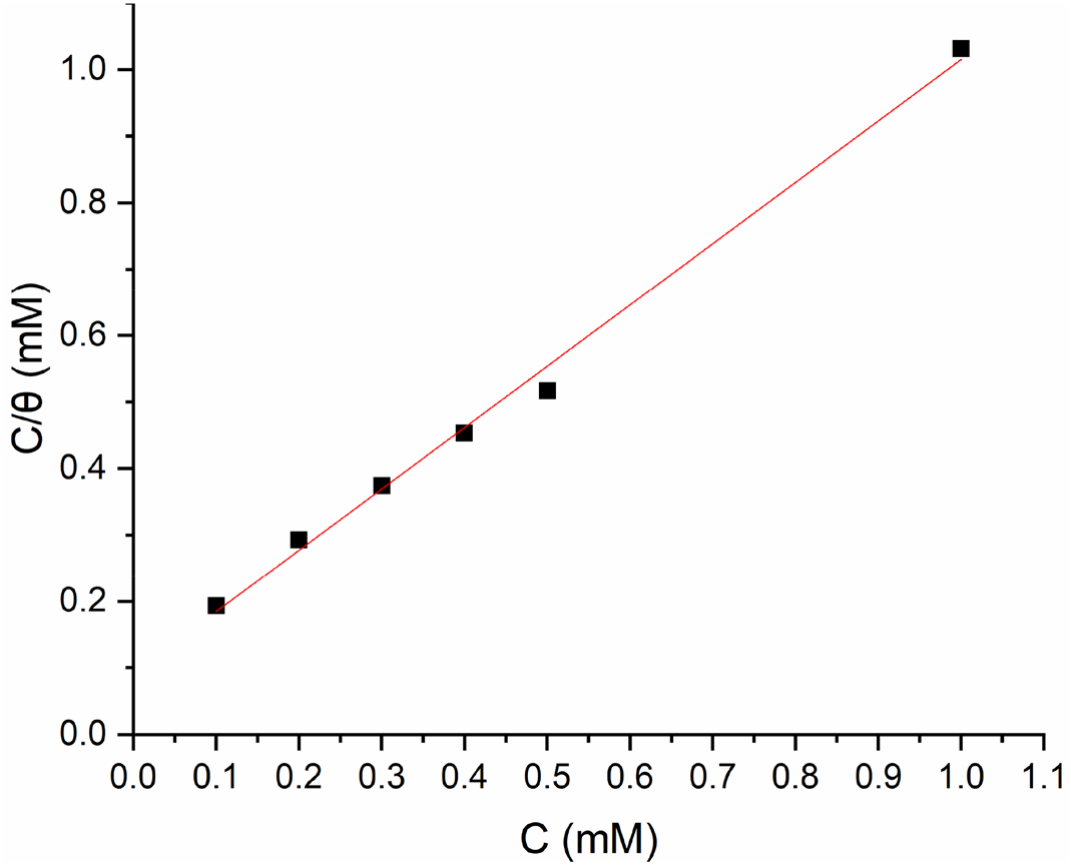
Langmuir adsorption isotherm for mild steel in presence of various concentrations of OHB.

The adsorption temperature facilitates the understanding of the relationship between the inhibitor molecules and the coupon surface. The isotherm that best fits the data was determined using the surface coverage (θ), which was collected using weight loss techniques. Various adsorption isotherms (Temkin, Freundlich, and Langmuir isotherms) were used to study the adsorption process to determine whether OHB molecules adhere physically or chemically to the surface of the coupon. The Langmuir adsorption isotherm fits the data well, as indicated by the regression coefficient (R2) for OHB of 0.99528, with the calculated slope and intercept values for the Langmuir isotherms of 0.92289 ± 0.03176 and 0.09269 ± 0.01614, respectively. Figure 11 shows Eq. (17) and describes the isothermal plot of Langmuir absorption between C/θ and C:

17 $$\frac{C}{\theta }=\frac{1}{{K}_{ads}}+C$$

where ${K}_{ads}$ is the equilibrium constant, stands for surface area, and $C$ is the OHB concentration.

The free energy of adsorption, $\Delta {G}_{ads}^{o}$ was calculated using the ${K}_{ads}$ value and a linear straight-fitted plot between $C/\theta$ and $C$. Equation (18) establishes a connection between ${K}_{ads}$ and $\Delta {G}_{ads}^{o}$:

18 $$\Delta {G}_{ads}^{o}=-RT ln\left(55.5{K}_{ads}\right)$$

where 55.5 is the measure of water content, $R$ is the gas constant, and $T$ is the temperature. "$\Delta {G}_{ads}^{o}$" was obtained by adding "${K}_{ads}$" to the above formula.

Physisorption is suggested by $\Delta {G}_{ads}^{o}$ around or even less negative than $-20 kJ{mol}^{-1}$, while chemisorption is suggested by $\Delta {G}_{ads}^{o}$ varying from $-40 kJ{mol}^{-1}$ and a larger negative value^37,38^. The OHB $\Delta {G}_{ads}^{o}$ of $-33.89 kJ{mol}^{-1}$ indicates that there are two distinct modes of adsorption, physical and chemical.

### Potentiodynamic polarisation measurements

Tafel extrapolating, potentiodynamic observations, cyclic polarization, and linear polarization impedance are a few of the technologies used in polarization, with the Tafel extrapolation critically involved in corrosion. Any polarization impedance measurement can be used to assess the corrosion dynamics of thin films (and the possible coating layers) as a time function. The primary factor used to assess the effectiveness of protective coating kinetics is current density; the higher the current density, the poorer the electrochemical behaviour. Furthermore, low pitting potential and a narrow passive potential range associated with rapid corrosion rates suggest coating flaws and pores that permit electrolyte diffusion, which encourages the loss of the protective coating^39^. One of the popular polarization techniques for determining corrosion rates is Tafel extrapolation, which is a quicker and more appropriate test than the traditional weight-loss measurement. However, it is known that the corrosion rates determined by Tafel extrapolation of polarization curves are frequently different from those determined by weight loss. The fact that the Tafel equation can be used to explain a variety of corrosion-related reactions makes it possible to represent a mixed potential theory, which predicts the corrosion rate and potential per the kinetics and thermodynamics of all reactions occurring on an electrode surface. Since corrosion circumstances are often excluded from the reversible potentials for all processes, Tafel kinetics provides a correct explanation of corrosion kinetics for situations where mass transport restrictions are not taken into account. When a metallic electrode is submerged in a corrosive aqueous environment, anodic and catalytic reactions will happen spontaneously on the electrode surface, leading to electrode corrosion. The electrode's subsequent potential and the reversible or equilibrium potentials of each reaction occurring on the surface will not be comparable in this case.

**Figure 12:**
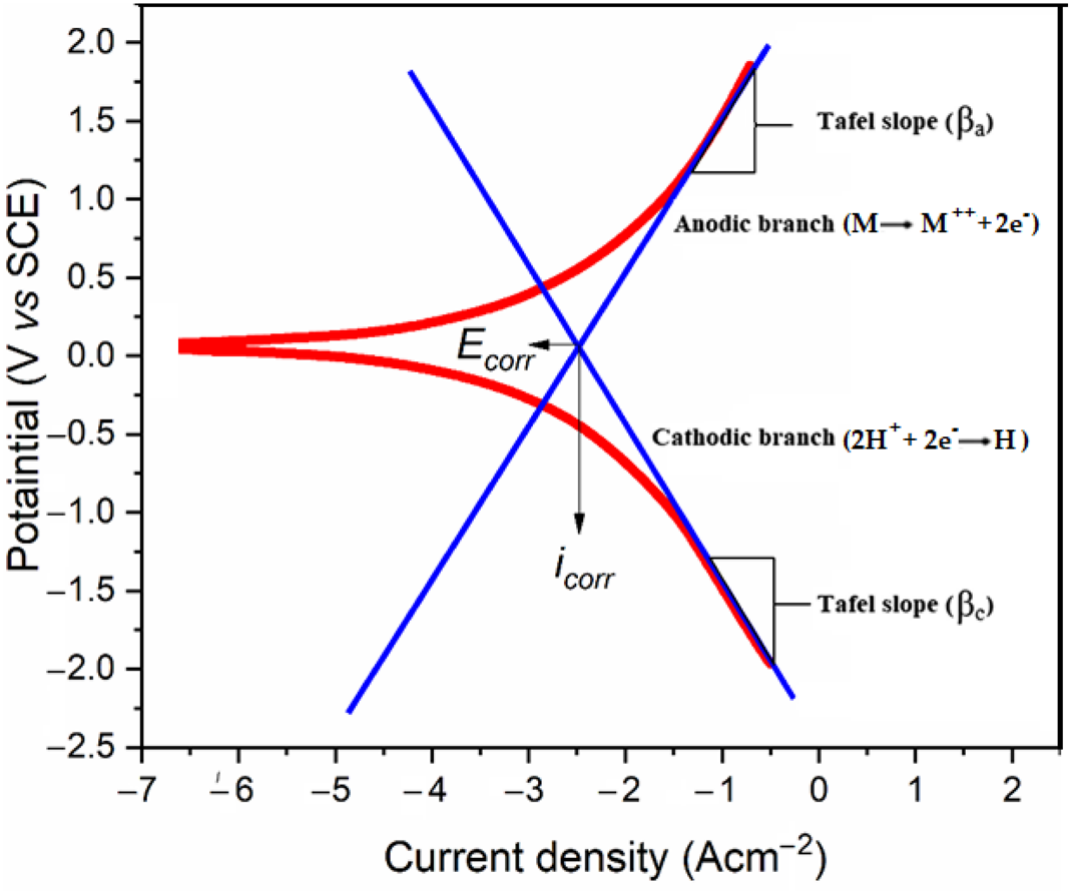
Through extrapolation, the Tafel slopes, corrosion potential, and corrosion current density were estimated.

The corrosion current density is obtained by extrapolating the linear component of the curve to E_corr_, as shown in Fig. 12. Under the assumption that corrosion is uniform, Faraday's law can be used to translate corrosion density into the rate of penetration or weight loss. This method makes it possible to monitor the system under study continuously in addition to measuring significantly lower corrosion rates.

Figure 12 demonstrates the method and Eq. (19) shows how the inhibitive efficacy was assessed:

19 $$\mathrm{IE}\left(\mathrm{\%}\right)=\frac{{\mathrm{i}}_{\mathrm{corr}}-{\mathrm{i}}_{\mathrm{corr}(\mathrm{inh})}}{{\mathrm{i}}_{\mathrm{corr}}}\times 100$$

where ${i}_{corr(inh)}$ is the current density with the addition of OHB whereas ${i}_{corr}$ represents the current density without the addition of OHB.

**Figure 13:**
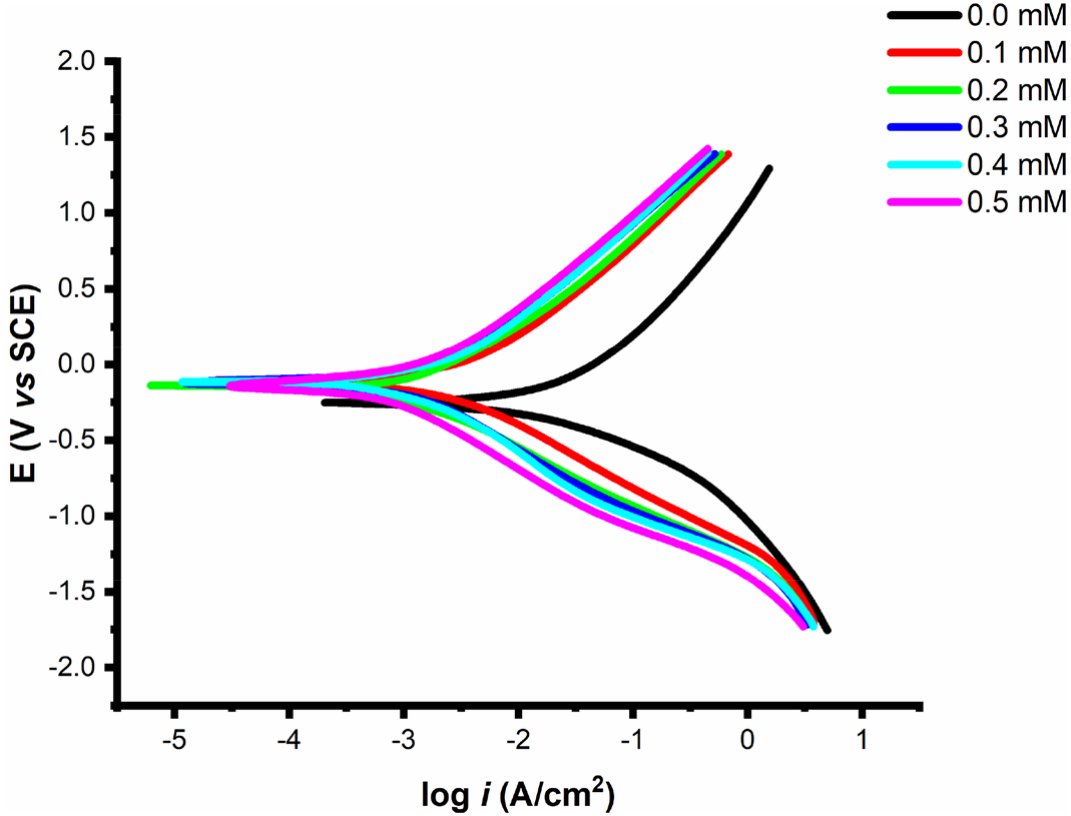
Mild steel polarization curves in 1 M HCl solution without and with the addition of different concentrations of OHB.

The polarization curves for the mild steel samples in 1.0 M HCl solution in both the absence and presence of different OHB concentrations are shown in Fig. 13 at 303 K. With the anodic (${\beta }_{a}$) and cathodic (${\beta }_{c}$) Tafel slopes, Table 3 shows the data for the corrosion potential (${E}_{corr}$), corrosion current density (${i}_{corr}$), and inhibitory efficiency. The Tafel fit technique which employs a nonlinear chi-square minimization to fit the data to the Stern-Geary equation is presented by the Gamry-E chem Analyzer application.

**Table 3:** Mild steel Tafel parameters in 1 M HCl solution without and with the addition of different concentrations of OHB.

*Conc. mM*	*E*_corr_ (mV)	β_a_ (mV/dec)	β_c_ (mV/dec)	*i*_corr_ (μA·cm^−2^)	*IE* (%)
0.0	− 480	245	215	510 ± 3.75	0
0.1	− 520	110.6	178.4	343.9 ± 3.90	68.9
0.2	− 528	97.4	144.6	200.8 ± 2.68	79.6
0.3	− 534	91.2	114.7	85.7 ± 1.88	88.3
0.4	− 524	74.8	107.8	67.7 ± 1.68	93.6
0.5	− 537	80.3	101.3	61.3 ± 2.15	96.5

When the ${\mathrm{E}}_{\mathrm{corr}}$ shift hits 85 mV, the corrosion inhibitor can be categorized as either cathodic or anodic. The molecules can be viewed as mixed-type since OHB displaces the majority of ${\mathrm{E}}_{\mathrm{corr}}$. The cathodic hydrogen evolution is delayed and the anodic dissolving of steel is slowed down when OHB is added to the corrosive solution. The corrosion rate dropped as the OHB concentration increased, boosting the inhibitory efficacy, as seen in Table 3, where the presence of OHB caused a decrease in ${\mathrm{i}}_{\mathrm{corr}}$ values. The OHB was in control of both processes, as evidenced by the Tafel constants (${\beta }_{a}$, ${\beta }_{c}$) rarely changing in the presence of the OHB. Adsorbed molecules had no impact on hydrogen evolution or mild steel dissolving^40^.

OHB effectively prevented mild steel from corroding in an acidic environment. It is also important to note that the corrosion potentials (E_corr_) shifted to a cathodic orientation with the addition of OHB, and the drop of the cathode branch is noticeably higher than that of the anode branch, therefore OHB suppresses the mild steel's cathodic reaction more than the anodic reaction^41^. The mild steel anode and cathode reactions in HCl are as follows^42^. Equation (20) describes the mechanism of cathodic reaction.

20 $${2H}^{+}+{2e}^{-}={H}_{2}\uparrow $$

The mechanism of anodic reaction in absence of OHB (Eqs. 21–24):

21 $$Fe+{Cl}^{-}\leftrightarrow ({FeCl}^{-}{)}_{ads}$$

22 $$({FeCl}^{-}{)}_{ads}\leftrightarrow (FeCl{)}_{ads}+{e}^{-}$$

23 $$(FeCl{)}_{ads}\leftrightarrow {FeCl}^{+}+{e}^{-}$$

24 $${FeCl}^{+}+{e}^{-}\leftrightarrow {Fe}^{++}+{Cl}^{-}$$

The mechanism of anodic reaction in presence of OHB (Eqs. 25–30):

25 $$Fe\cdot {H}_{2}{O}_{ads}+inh\leftrightarrow {FeOH}_{ads}^{-}+{H}^{+}+inh$$

26 $$Fe\cdot {H}_{2}{O}_{ads}+inh\leftrightarrow {Fe\cdot inh}_{ads}+{H}_{2}O$$

27 $${FeOH}_{ads}^{-}\to {FeOH}_{ads}+{e}^{-}$$

28 $${Fe\cdot inh}_{ads}\leftrightarrow {Fe\cdot inh}_{ads}^{+}+{e}^{-}$$

29 $${{FeOH}_{ads}+Fe\cdot inh}_{ads}^{+}\leftrightarrow {FeOH}^{+}+{Fe\cdot inh}_{ads}$$

30 $${FeOH}^{+}+{H}^{+}\leftrightarrow {Fe}^{+}+{H}_{2}O$$

The addition of OHB creates a $Fe\cdot {inh}_{ads}$ blocking layer on the mild steel surface, effectively preventing the precipitation of anodic iron ions, and decreasing the charge at the mild steel/solution interface, which severely restricts the precipitation of the cathodic hydrogen reaction.

### Electrochemical measurements

**Figure 14:**
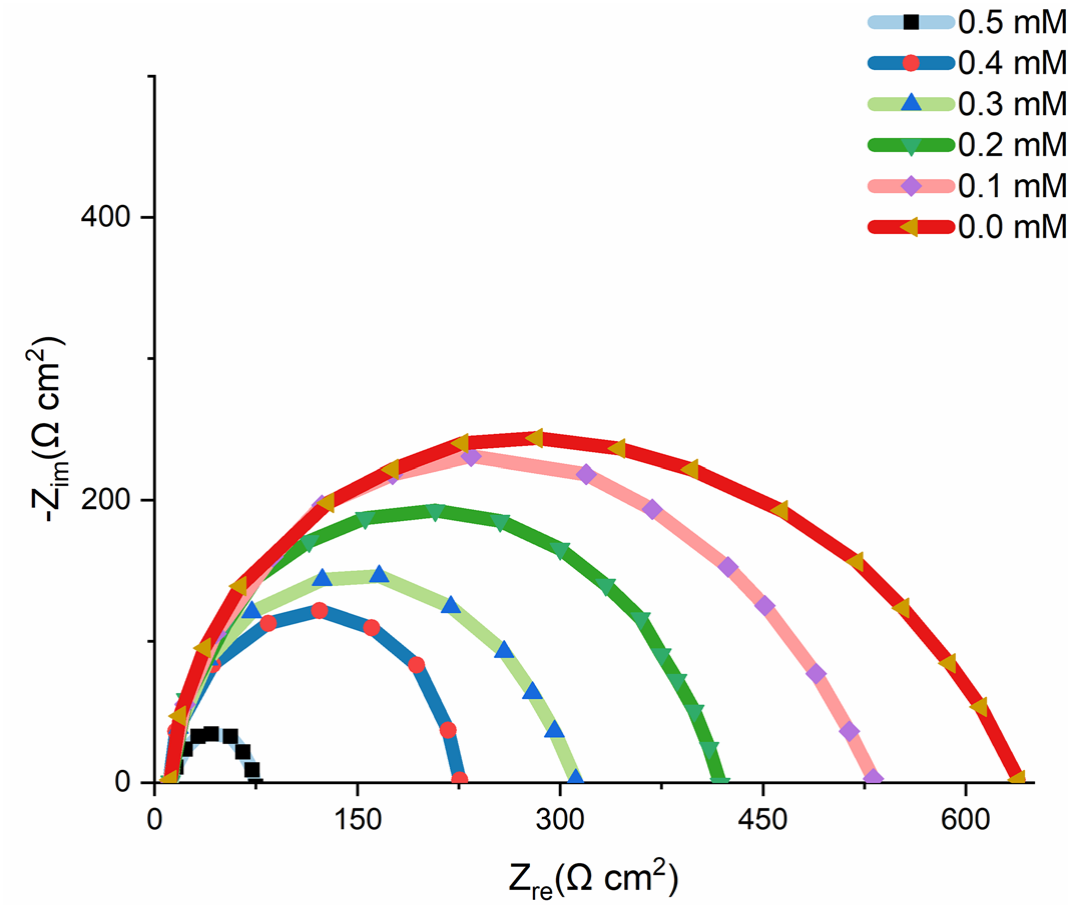
Mild steel Nyquist plots in 1 M HCl solution without and with the addition of different concentrations of OHB.

The effectiveness of the OHB at preventing corrosion was assessed using electrochemical impedance spectroscopy (EIS). Table 4 displays the corrosion data at 303 K without and with the addition of OHB, and Fig. 14 displays the Nyquist plots. The total resistivity of mild steel in HCL was dramatically increased by the presence of OHB. With less inductive action at lower frequencies, there are two loops in the Nyquist plots: one in the high-frequency band (HF) and one at an intermediate frequency (MF) (LF). Thus, the HF and MF loops are related to charge-transfer mechanisms and the EIS instrument limitations at high frequencies with low impedance. Therefore, the relaxing process of the corrosion product adsorption or the OHB molecule adsorption onto the specimen surface in a corrosive solution without or with the addition of OHB, respectively, is responsible for the inductive behaviour observed in the LF area^43^.

**Table 4:** Mild steel EIS parameters in 1 M HCl solution without and with the addition of different concentrations of OHB.

Conc. (mM)	Rs (Ω cm^2^)	Rct (Ω cm^2^)	Cdl (μF)	IE%
0.0	2.4	55.85	530	0
0.1	2.3	104.84	285	65.7
0.2	2.2	213.24	305	74.6
0.3	2.4	309.55	230	81.8
0.4	2.4	395.67	175	90.5
0.5	2.3	512.88	122	96.1

Equation (31) was used to obtain the inhibition efficiency (IE%) from the charge-transfer impedance:

31 $$IE\left(\mathrm{\%}\right)=\frac{{R}_{ct}^{\mathrm{^{\prime}}} - {R}_{ct}}{{R}_{ct}^{\mathrm{^{\prime}}}}\times 100$$

where ${R}_{ct}^{\mathrm{^{\prime}}}$ and ${R}_{ct}$ are the charge-transfer-resistance without and with the addition of OHB.

According to Table 4, the charge-transfer resistance (${R}_{ct}$) increased as the concentration of OHB increased, eventually corroding the system because of the high charge-transfer impedance^44^. Additionally, a lower mild steel capacitance is related to a higher inhibitor resistance. The OHB adsorbed into the material's interface with the solution based on the observed rise in the ${C}_{dl}$ is associated with an increase in the local dielectric constant and/or the thickness of the electrical double layer^45^.

Figure 14 illustrates how Nyquist diagrams reveal incomplete capacitive rings. A higher corrosion rate is consistently proved at larger ring radii. When OHB is introduced, the breadth increases, demonstrating that it has an inhibitory effect on mild steel corrosion in the test solution. This results from the inhibitor molecules adhering to and producing a shield-like layer on the mild steel surface. The ${C}_{dl}$ double layer's lower estimated capacitance suggests that the film coating the surface reduces corrosion^46^.

**Figure 15:**
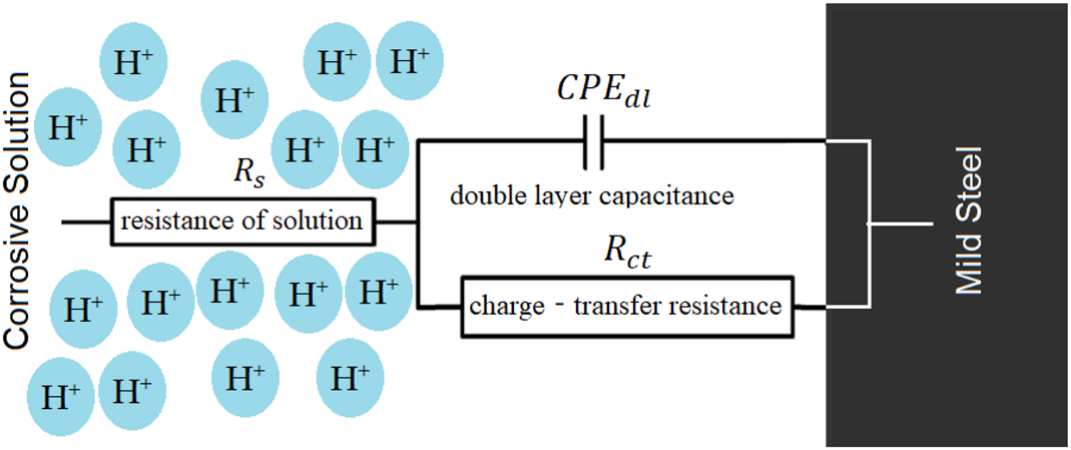
EIS findings which were fitted using the equivalent circuit model.

The adsorption of the OHB on the most active adsorption centres may be responsible for the observed increase in the ${C}_{dl}$ in the acidic medium with the addition of OHB^47^. The homogeneity of the absorbed OHB layer was reduced by the corrosion. In addition, the IE% followed a similar trend as the inhibition efficacy determined by the dynamic efficacy approach and mass loss with increasing inhibitor concentration. The equivalent circuit diagram of the EIS equation for corrosive environments without and with the addition of the inhibitor can be seen in Fig. 15.

The charge transfer resistance rating was used to determine how well the electrons crossed the contact. The components of the circuit are solution resistance (${R}_{s}$), stationary phase element (${CPE}_{dl}$), and charge transfer resistance (${R}_{ct}$)^48^.

A complex nonlinear least squares (CNLS) simulation was utilized since the simulated values were calculated using an equivalent circuit and compared to actual data.

### Surface morphology

**Figure 16:**
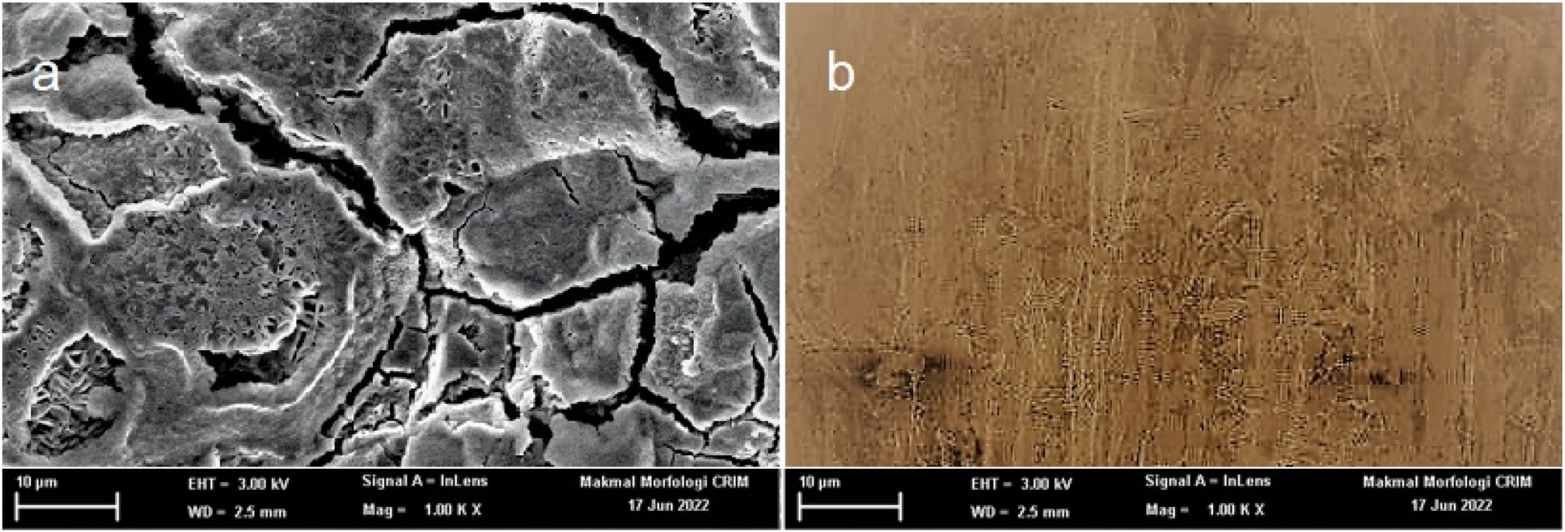
Mild steel SEM photographs in 1 M HCl solution without (**a**), and with (**b**) the addition of different concentrations of OHB.

Figure 16 a and b shows SEM images of the mild steel surface coupon after 5 h of exposure to 1 M HCl with and without the addition of OHB. The surface shape was drooping and crowning, as shown in Fig. 16a, indicating significant corrosion of the mild steel surface. Figure 16b shows the surface morphology in the presence of OHB with significantly decreased corrosion compared to Fig. 16a.

### Theoretical calculations

**Figure 17:**
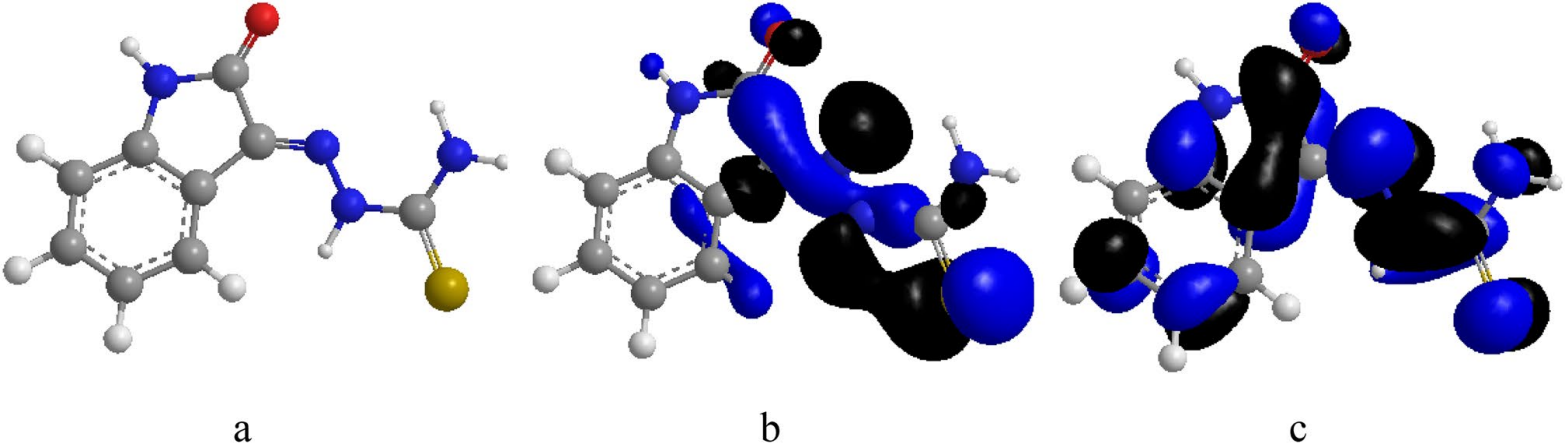
OHB molecular structure, HOMO and LUMO at B3LYP/6-31G(d,p) level.

Frontier molecular orbitals (FMOs), the Highest Occupied molecular orbital (HOMO) which is the highest-energy molecular orbital that has electrons in it and the Lowest Unoccupied molecular orbital (LUMO), that is, the lowest-energy molecular orbital that does not have any electrons in it are very important for reactivity. The potential to give an electron is represented by the HOMO, which has electrons, while the LUMO represents the potential to receive an electron, which is unoccupied and acts as an electron acceptor. The kinetic stability, chemical reactivity, optical polarizability, and chemical hardness–softness of a molecule are determined by the energy gap between HOMO and LUMO^49^. The HOMO and LUMO orbital energies in this work were initially computed using the B3LYP technique with 6-31G (d,p). The findings and additional hypotheses were used in all other computations. While ELUMO denotes a molecule's ability to accept an electron, greater levels of EHOMO suggest an increase in the electron donor, which results in good inhibitory action with increasing adsorption of the inhibitor on a metal substrate. The ability of the inhibitor molecules to be adsorbed on the mild steel surface increases as EHOMO increases and ELUMO decreases. While the appearance of the three-dimensional structure of the OHB molecule is demonstrated in Fig. 17, the HOMO and LUMO energies are displayed in Table 5.

**Table 5:** Computation of the investigated inhibitor OHB's quantum chemical properties in the gaseous phase at B3LYP/6-31G(d,p).

${E}_{\mathrm{HOMO}}$	${E}_{\mathrm{LUMO}}$	$\Delta E$	$I$	$A$	$\chi$	$\eta$	$\sigma$	$\Delta N$	$\upomega$
− 8.927	− 2.784	− 6.143	8.927	2.784	5.8555	3.0715	0.3255	0.1863	5.580

From the observation of the aforementioned theoretical findings, it appears that the molecular structure of OHB is well-organized and very well-adsorbed on the mild steel surface, achieving a large coverage surface area (i.e. high corrosion inhibition), which is in good agreement with the findings from the experimental techniques. From the energy-optimized molecular geometry for OHB, it is seen that the thiosemicarbazone side chain protrudes out of the plane while the benzene ring and thiazole ring remain planar (Fig. 17a). The benzene and thiazole rings are excellently suited for adsorption on the mild steel surface due to their planar orientation. The electronic distributions in HOMO are spread over these two rings and the thiosemicarbazone side chain as shown in Fig. 17b. Furthermore, Fig. 17c shows that the electronic distributions in LUMO are scattered along the thiosemicarbazone side chain, making it easier for electrons to move from the inhibitor molecules' HOMO to unoccupied iron 3d orbitals and from the full 4 s iron orbital to the LUMO^50^. The rate of the anodic metal dissolution is slowed down by the transfer of electrons from inhibitor molecules, which raises the electron density on the anodic sites of mild steel. However, retro-electron donation results in a lack of electron density at the cathodic locations where hydrogen ions are electrocuted (cathodic hydrogen evolution reaction). Thus, the exhibited mixed-type inhibitory features of the OHB molecules are explained by simple two-way electrical transfer. In addition to adhering to the surface of mild steel, OHB molecules bind iron atoms to create a metal complex, leading to a passive iron ion-inhibitor film developing on the mild steel surface inhibiting additional oxidation. Despite not being directly engaged in charge transfer during adsorption, the thiosemicarbazone side chain may have a significant impact on the entire adsorption mechanism. In general, higher HOMO energy indicates that electron donation at this level is easy, while lower LUMO energy indicates that electron acceptance is easy. Therefore, narrowing the energy gap indicates a positive interaction between the donor–acceptor system through forward and backward electron donation. In the present case, it is observed that the HOMO energy is high and the LUMO energy is relatively low. Despite the energy gap generally widening, the effect of electron donation from the inhibitor to the metal is relatively more widespread than that of the retroactive donation from the metal to the inhibitor. The Electronic Donation portion ($\Delta$ N) also reflects this. Elnga et al. claimed that when $\Delta$ N is a positive number, the inhibition efficacy of the corrosion inhibitor increases due to electron donation to the metal^51^, which is assisted by the low electronegativity value ($\chi$) of OHB. OHB can engage in more complicated interactions with other molecules when their global softness ($\sigma$) is higher. Another crucial factor that contributes to improved electrostatic contact between two interacting systems is the dipole moment. A higher OHB dipole moment indicates a more powerful electrostatic interaction between OHB and the charged metal surface. OHBs with more substituent groups have larger molar volumes and can cover more surface area as a result. The opposite of electrophilicity is called nucleophilicity $(\varepsilon ={\omega }^{-1}$). The $\omega$ depicts an inhibitor molecule's propensity for accepting an electron, thus, a good nucleophile is characterized by a low $\omega$ (and hence a low $\chi$), and vice versa.

### Mulliken Charges

It is common practice to use Mulliken charges to measure atomic charges within molecules and locate inhibitor adsorption sites. Furthermore, the negative charge of a heteroatom grows with its capacity for donor–acceptor adsorption onto a metallic substrate. The two nitrogen and oxygen atoms have high atomic charges (N(3) = − 0.3344, N(15) = − 0.3517, O(12) = − 0.2946, N(1) = − 0.2749, and S(14) = − 0.2697), which suggests that they are in charge of iron absorption. Table 6 shows the atomic charges of the inhibitor compounds.

**Table 6:** Mulliken atomic charges for the corrosion inhibitor.


Atoms	Charges	Atoms	Charges	Atoms	Charges	Atoms	Charges
N(1)	− 0.2749	C(5)	− 0.1057	C(9)	− 0.0798	C(13)	0.1780
N(2)	0.0219	C(6)	− 0.1300	C(10)	− 0.1700	S(14)	− 0.2697
N(3)	− 0.3344	C(7)	− 0.0661	C(11)	0.0867	N(15)	− 0.3517
C(4)	0.3640	C(8)	− 0.1637	O(12)	− 0.2946	H(16)	0.2599

### Mechanism of inhibition

**Figure 18:**
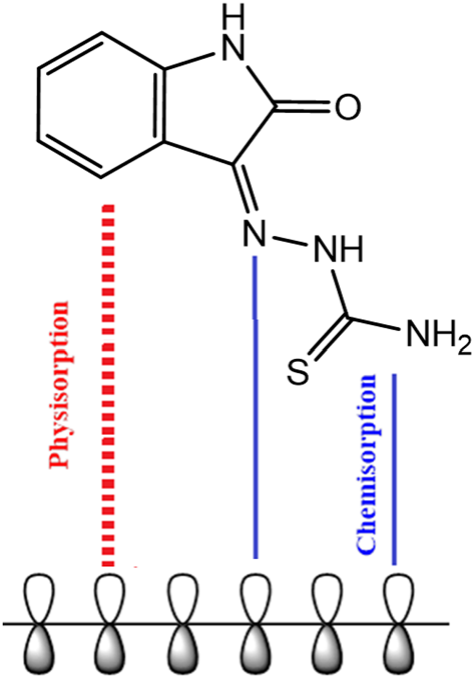
The proposed mechanism of corrosion inhibition of mild steel in 1 M HCl.

The chemical structure, charge, behaviour of the acidic environment and surface characteristics of the metallic surface all impact how the inhibitor molecules adsorb onto the surface. Inhibitors enable metals to be absorbed from aqueous solutions by suppressing the active sites on the metal that are vulnerable to corrosion. The creation of a defensive barrier that is adsorbed onto the metallic substrate gives organic molecules their inhibitory resistance. Measurements of weight loss and electrochemical methods showed that the inhibitor significantly decreased mild steel corrosion. Additionally, the Langmuir adsorption model's predictions for how the inhibitor molecules stick to the mild steel surface were closely supported by the adsorption isotherm investigations. Electrostatic interactions with protonated heteroatoms and different connections between inhibitor molecules impact how the protective coating adheres to the mild steel surface^52^. Numerous heteroatoms, pi-bonds, aromatic rings with lone pairs of electrons, and the investigated inhibitor molecule all help to form coordination bonds and significantly increase the amount of adsorption on the mild steel surface. According to the free energy parameter, the interaction between iron d-orbitals and the studied inhibitor compounds generally followed chemical adsorption. Heteroatoms with electron pairs in the OHB have encouraged chemisorption onto the mild steel surface. The hypothesized mechanism for mild steel corrosion inhibition in a corrosive media is shown in Fig. 18.

## Conclusions

Due to the existence of extremely effective electronic adsorption centres (O, N, S, and pi-bonds) that block the metal active sites, OHB exhibits significant corrosion protection for mild steel in 1 M HCl. The key findings are as follows:

- The synthesized OHB exhibits good mild steel corrosion inhibition efficiency in a 1 M HCl environment. The inhibition efficiency increases with increasing OHB concentration and decreases with rising temperature.
- OHB participates in chemical adsorption on metallic surfaces and weakly binds to the metal surface, with the inhibitory efficacy decreasing as the temperature rises. The maximum inhibition efficiency was 96.7% at 303 K in 1 M HCl solution.
- The formation of a protective layer of inhibitor molecules at the steel-electrolyte interface is the method by which OHB inhibits mild steel corrosion.
- The Gads model indicates chemisorption and physisorption, and the adsorption mechanism is spontaneous.
- According to the quantum chemical simulations, OHB adsorbs onto a mild steel surface using oxygen, sulphur, and nitrogen.
